# A Comprehensive Method for Accurate Strain Distribution Measurement of Cell Substrate Subjected to Large Deformation

**DOI:** 10.1155/2018/8504273

**Published:** 2018-01-08

**Authors:** Hong He, Rong Zhou, Yuanwen Zou, Xuejin Huang, Jinchuan Li

**Affiliations:** ^1^College of Materials Science and Engineering, Sichuan University, Chengdu 610065, China; ^2^College of Architecture and Environment, Sichuan University, Chengdu 610065, China

## Abstract

Cell mechanical stretching *in vitro* is a fundamental technique commonly used in cardiovascular mechanobiology research. Accordingly, it is crucial to measure the accurate strain field of cell substrate under different strains. Digital image correlation (DIC) is a widely used measurement technique, which is able to obtain the accurate displacement and strain distribution. However, the traditional DIC algorithm used in digital image correlation engine (DICe) cannot obtain accurate result when utilized in large strain measurement. In this paper, an improved method aiming to acquire accurate strain distribution of substrate in large deformation was proposed, to evaluate the effect and accuracy, based on numerical experiments. The results showed that this method was effective and highly accurate. Then, we carried out uniaxial substrate stretching experiments and applied our method to measure strain distribution of the substrate. The proposed method could obtain accurate strain distribution of substrate film during large stretching, which would allow researchers to adequately describe the response of cells to different strains of substrate.

## 1. Introduction

Cardiovascular mechanobiology [1–5] is a discipline that focuses on the effects of the mechanical environment on the cardiovascular system and elucidates how mechanical factors produce biological effects that lead to vascular remodeling. Cardiovascular mechanobiology aims to provide biomechanical solutions for the diagnosis, prevention, and rehabilitation of cardiovascular disease. Cell mechanical stretching *in vitro*, vascular stretching *in vitro*, an animal model of stress changes in blood vessel *in vivo*, and numerical simulation method are commonly used in cardiovascular mechanobiology experiments. Cell-substrate stretching technique, extensively used *in vitro* cell mechanical experiments, is an effective method of force transduction [6–8]. Riehl et al. [9] summarized the methods of mechanical stretching in cell-substrate stretching experiments and showed that most of the strain loaded on substrate was large, with the maximum value up to 33%. Besides, strain distribution varies from region to region within a substrate. Thus, it is of great importance to analyze the accurate strain field of substrate under large deformation.

Some commonly used methods for measuring substrate strain include the direct calculation method, the resistance strain gauge measuring method, the phase shift shadow moiré method, and the finite element analysis. In the direct calculation method [10], several marks should be drawn first on the surface of substrate, so that the displacement and strain can be calculated by analyzing the difference of those marks between the images captured before and after deformation. The accuracy of this method is heavily dependent on the distribution of marks. Therefore, the direct calculation method is a rough measurement. The resistance strain gauge measurement method [11] attaches strain sensors to the surface of substrate for strain measurement. However, the use of any contact-type sensor would obstruct the stretching of substrate especially under large deformation and lead to unexpected results. The phase shift shadow moiré method [12] requires complex operation, resulting in large systematic errors, since its accuracy would be influenced by light intensity. The finite element analysis [13, 14] is a computational simulation method, which can investigate many mechanical properties such as displacement and strain by setting material parameters and environmental parameters. However, the finite element analysis is usually based on models under ideal assumptions and cannot replace the actual experiment.

Digital image correlation (DIC) is a contactless full-field displacement and strain measurement technique. Since it was first proposed in the 1980s [15, 16], DIC is now extensively applied in many experimental mechanics researches [17, 18]. Digital image correlation engine (DICe) [19], developed by the Sandia National Laboratory, is an open-source library of DIC. With DICe, DIC would become a convenient and user friendly tool so that users can pay more attention to their researches rather than repeatedly programming the basic and complex procedures of DIC. To the best of our knowledge, to date, DICe has not been used to obtain accurate strain field of substrate in cell-substrate stretching experiments under large strain. The Newton-Raphson (NR) algorithm was used in DICe to calculate the desired deformation. However, in NR algorithm, the calculation result of the previous step is regarded as the start value of iteration for the next step. So the initial guess is of great importance in calculating strain field, especially under large deformation. An unreliable initial guess would seriously impact the results of strain measurement. Pan et al. [20] developed an incremental reliability-guided DIC technique (RG-DIC) for large deformation measurement, in which the recently developed robust RG-DIC technique was combined with an automatic reference image updating scheme, and the reference image for DIC analysis is automatically updated according to the seed point's zero-mean normalized cross-correlation (ZNCC) coefficient. This method could deal with specimens with irregular geometric shape and/or subjected to discontinuous deformation as well as minimize the accumulated errors in finally estimated displacements. Zhou et al. [21] proposed a fully automated method. In this method, the computer vision technique was used to extract image feature points and to match them between reference and deformed images. The deformation parameters of the seed point are initialized from the affine transform, and then the refined parameters are automatically transferred to adjacent points using a modified quality-guided initial guess propagation scheme. This method can accurately initialize all points of interest for the deformed images even in the presence of large rotation and/or heterogeneous deformation. Zhao et al. [22] proposed the utilization of three well-known population-based intelligent algorithms (PIAs), that is, genetic algorithm (GA), differential evolution (DE), and particle swarm optimization (PSO), and then incorporated standard PIAs with three improving strategies, including Q-stage evolutionary (T), parameter control (C), and space expanding (E) strategies, finally derived of a total of eighteen PIA-based algorithms. They tested these algorithms and found that DE-TCE performed best. Another large deformation measurement scheme, combining improved coarse search method and updating reference image scheme, was proposed by Tang et al. [23]. With this method, not only extremely large deformation can be measured successfully but also the accumulated error introduced by updating reference image could be controlled. Pan et al. [24] proposed an integrated scheme which combined the Fourier–Mellin transform-based cross-correlation (FMT-CC) for seed point initiation with a reliability-guided displacement tracking (RGDT) strategy for the remaining points. This method can provide an accurate initial guess for DIC calculation, even in the presence of large rotations. In order to obtain reliable initial values and then calculate accurate strain field of cell substrate under large strain, a simple method was proposed in this study and was combined with DICe. Compared with methods described above, without the use of feature detection, feature point matching, and other techniques, our method can accurately obtain the initial guess of the NR iteration for large deformation.

## 2. Materials and Methods

### 2.1. DIC Principles

#### 2.1.1. Basic Principles

DIC is an optical-numerical full-field displacement measuring technique with subpixel accuracy. In its basic principle, the measurement is performed by tracking or matching the same points (or pixels) between the two images recorded before and after deformation [17, 25]. The image recorded before and after motion is called reference image and deformed image, respectively. In general, the calculation area, also called the region of interest (ROI), should be specified or defined in the reference image, which is further divided into evenly spaced virtual grids. Moreover, each intersection point of virtual grids is selected as the center of a subset. The subset is the matching unit within the ROI, usually a square of (2*M* + 1) × (2*M* + 1) pixels. Then, a correlation criterion should be defined to evaluate the degree of similarity between reference and deformed subsets. The matching procedure is completed through searching the peak position of the distribution of correlation coefficient. Once the correlation coefficient extremum is detected, the position of the deformed subset is determined. The differences in the positions of the reference subset center and the target subset center yield the in-plane displacement vector at the calculate point.

#### 2.1.2. Shape Function

Based on the assumption of deformation continuity of a deformed solid object, a set of neighboring points in a reference subset remains as neighboring points in the target subset. The coordinate of points around the subset center in the reference subset can be mapped to points in the target subset according to the shape function. The first-order shape function that allows translation, rotation, shear, normal strains, and their combinations of the subset is most commonly used.

(1) $$\begin{array}{c} x_{i}^{\prime }=x_{i}+u+u_{x}\Delta x+u_{y}\Delta y, \end{array}$$

(2) $$\begin{array}{c} y_{j}^{\prime }=y_{j}+v+v_{x}\Delta x+v_{y}\Delta y, \end{array}$$

where *x*_*i*_, *y*_*j*_ is the local coordinate of each pixel point in reference subset, *x*_*i*_′, *y*_*j*_′ is the coordinate in the deformed subset, *u* and *v* are the displacement components, and *u*_*x*_, *u*_*y*_, *v*_*x*_, and *v*_*y*_ are the displacement gradient components.

#### 2.1.3. Correlation Criterion

To evaluate the similarity degree between the reference and deformed subsets, a correlation criterion should be defined in advance before correlation analysis. It is concluded that the zero-normalized cross-correlation (ZNCC) or zero-normalized sum of squared differences (ZNSSD) correlation criterion offers the most robust noise-proof performance and is insensitive to the offset and linear scale in illumination lighting [17].

#### 2.1.4. Subpixel Registration Algorithm

As shown in (1) and (2), the coordinate of points in the deformed subset may locate between pixels. Thus, before evaluating the similarity between reference and deformed subsets using the correlation criterion, the reference image and deformed image must be reconstructed by a certain kind of subpixel registration algorithm to further improve the accuracy of DIC.

#### 2.1.5. DICe

DICe is an open-source library of DIC, which can be used as a module in an external application or as a standalone analysis code. With DICe, DIC would become a more convenient tool and decrease the complexity when users apply it to their researches.

In DICe, the ZNSSD [17] is applied as the correlation criterion for its insensitivity to offset and linear scale in illumination lighting.

(3) $$\begin{array}{c} C_{ZNSSD}=\overset{N}{\underset{i=-N}{\sum }}\overset{N}{\underset{j=-N}{\sum }}{\left[\frac{f\left(x_{i},y_{j}\right)-f_{m}}{\sqrt{\sum_{i=-N}^{N}\sum_{j=-N}^{N}{\left[f\left(x_{i},y_{j}\right)-f_{m}\right]}^{2}}}-\frac{g\left(x_{i}^{\prime },y_{j}^{\prime }\right)-g_{m}}{\sqrt{\sum_{i=-N}^{N}\sum_{j=-N}^{N}{\left[g\left(x_{i}^{\prime },y_{j}^{\prime }\right)-g_{m}\right]}^{2}}}\right]}^{2}, \end{array}$$

where *f*(*x*_*i*_, *y*_*j*_) and *g*(*x*_*i*_′, *y*_*j*_′) denote the grayscale level of each point in the reference and deformed images, respectively, and *f*_*m*_ and *g*_*m*_ are the mean intensity value of two subsets. *x*_*i*_, *y*_*j*_ is the local coordinate of each pixel point in reference subset, and *x*_*i*_′, *y*_*j*_′ is the coordinate in the deformed subset.

As a classic algorithm in DIC, the NR algorithm is utilized in DICe as the iterative spatial domain cross-correlation algorithm. In the first-order shape function, the desired mapping parameter vector is **p** = (*u*, *v*, *u*_*x*_, *u*_*y*_, *v*_*x*_, *v*_*y*_)^*T*^, where *u*, *v* denotes the displacement components and *u*_*x*_, *u*_*y*_, *v*_*x*_, *v*_*y*_ is the strain of subset. To acquire the desired deformation vector by the NR iteration method, the solution can be written as [17]

(4) $$\begin{array}{c} p=p_{0}-\frac{\nabla C\left(p_{0}\right)}{\nabla \nabla C\left(p_{0}\right)}, \end{array}$$

where **p**_0_ is the initial guess of the solution, **p** is the next iterative approximation solution, ∇*C*(**p**_0_) is the gradients of correlation criteria, and ∇∇*C*(**p**_0_) is the second-order derivation of correlation criteria.

### 2.2. Improved DIC Scheme

**Figure 1:**
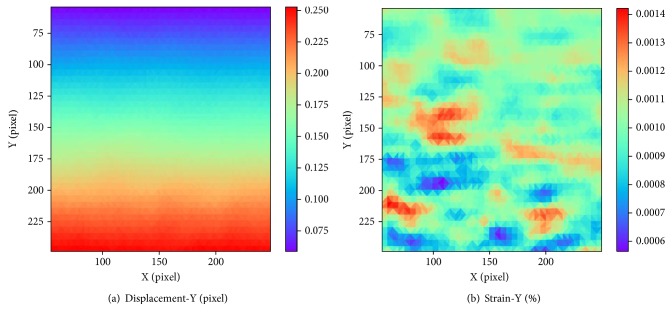
Calculation results by DICe under 0.1% strain: (a) displacement distribution along *y*-axis; (b) strain distribution along *y*-axis.

**Figure 2:**
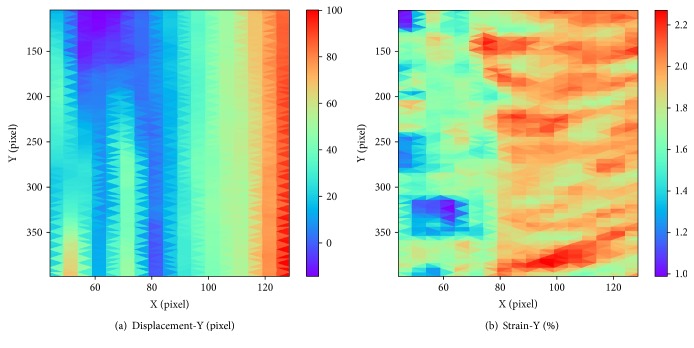
Calculation results by DICe under 10% strain: (a) displacement distribution along *y*-axis; (b) strain distribution along *y*-axis.

As described above, the NR algorithm was used in DICe to calculate the desired deformation vector. However, when it is applied to large strain measurement directly, the iteration divergence would appear, so that the accurate strain cannot be obtained. Figures 1 and 2 show the calculation results of DICe under small and large strain, respectively.  Figure 1 is under 0.1% strain and Figure 2 is under 10% strain. As shown in Figure 1, the displacement distribution is continuous and the strain field is uniform. The strain distribution is very close to its setting value 0.1%, suggesting that the DICe calculation is valid and accurate. Nevertheless, as shown in Figure 2, the displacement distribution is not continuous which cannot happen in the actual, indicating that these results are invalid.

In traditional DICe, the coordinate (0,0) was used as the default NR iteration initial value of displacement (*u*_0_,*v*_0_), which is only suitable in small deformation situation. The iteration would not satisfy the converge condition when applied to calculating large strain. Therefore, the result in Figure 2 is caused by the default iterative initial value. Vendroux and Knauss [26] found that convergence range of the NR iteration is generally within 7 pixels through experimental results. However, the large deformation is common in many cardiovascular mechanobiology cell mechanical stretching experiments. Therefore, searching a reliable initial value is of great importance under large strain. In order to get the reliable initial guess, we proposed a comprehensive method and combined it with DICe.

**Figure 3:**
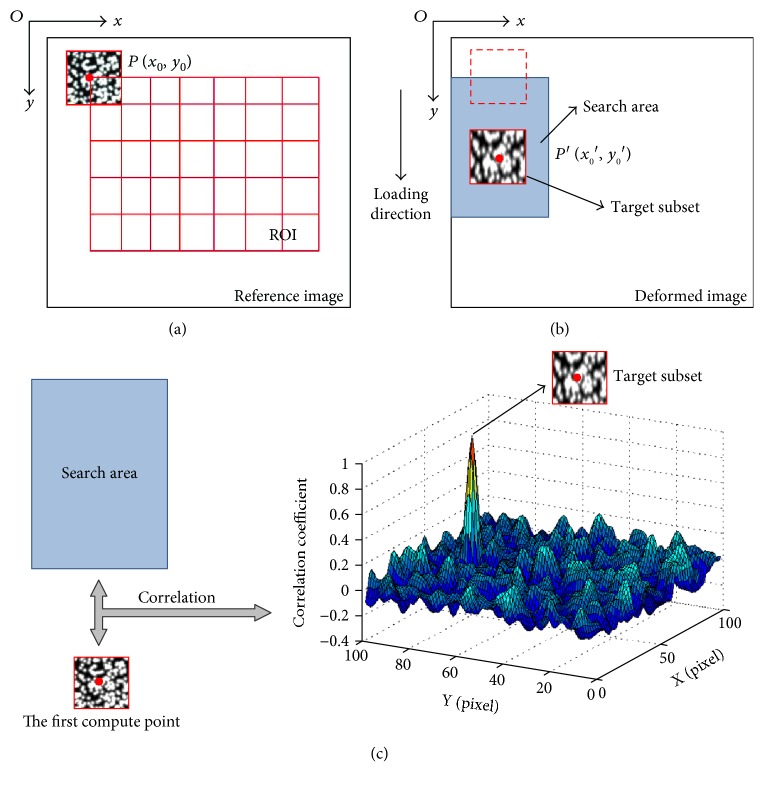
Schematic illustration of the proposed method: (a) reference image; (b) deformed image; (c) matching results.

To illustrate the proposed method, a schematic drawing is shown in Figure 3. Conventional correlation calculation generally starts with the upper-left point of the ROI, so the *P*(*x*_0_, *y*_0_) point in Figure 3(a) is the first calculation point. After a uniaxial loading, *P*(*x*_0_, *y*_0_) has moved to a new position called *P*′(*x*_0_′, *y*_0_′) as shown in Figure 3(b).

In order to get the reliable initial guess, a searching area along the deformation direction should be set. Then, the similarity degree between the first subset in the reference image and all subsets of the searching area in the deformed image should be calculated by the chosen correlation criterion. The subset with the optimal similarity degree and the first subset is the target subset.

(5) $$\begin{array}{c} C\left(p\right)=C\left(u,v\right)=\overset{M}{\underset{i=1}{\sum }}\overset{M}{\underset{j=1}{\sum }}{\left[\frac{f\left(x_{i},y_{j}\right)-f_{m}}{\sqrt{\sum_{i=1}^{M}\sum_{j=1}^{M}{\left[f\left(x_{i},y_{j}\right)-f_{m}\right]}^{2}}}-\frac{g\left(x_{i}+u,y_{j}+v\right)-g_{m}}{\sqrt{\sum_{i=1}^{M}\sum_{j=1}^{M}{\left[g\left(x_{i}+u,y_{j}+v\right)-g_{m}\right]}^{2}}}\right]}^{2}, \end{array}$$

where *u*, *v* denotes the displacement components, and *f*_*m*_ and *g*_*m*_ are the mean intensity value of two subsets, respectively. *M* is the size of the first subset, **p** = (*u*, *v*)^*T*^ is the displacement parameter vector, and *x*_*i*_, *y*_*j*_ is the coordinate of each pixel point in the first subset. It is worth noting that the points (*x*_*i*_ + *u*, *y*_*j*_ + *v*) in the deformed image should be located in the searching area. Then, the maximum value of *C*(*u*, *v*) would be calculated.

(6) $$\begin{array}{c} C\left(p^{\prime }\right)=\max\left(C\left(u,v\right)\right), \end{array}$$

where **p**′ is the displacement parameter vector corresponding to the maximum value. The parameters *u* and *v* in **p**′ are the initial guess for NR iteration.

The detailed process of the proposed method is as follows:

**Step 1**

In the reference image, define the ROI and divide it into evenly spaced virtual grids. Each intersection point of virtual grids is selected as the center of a subset.

**Step 2**

Reconstruct the deformed image. Choose fourth-order Keys interpolant (Keys4) algorithm as the subpixel registration algorithm.

**Step 3**

Set deformation direction, the size of the searching area. Obtain the reliable initial guess for NR iteration with the method described above. The calculated (*u*,*v*) is the initial guess for NR iteration in the next step.

**Step 4**

Choose the next calculate point and calculate its displacement and strain. The displacement and strain suppose to be calculated by NR iteration using the initial guess obtained in the last step. It is worth noting that the number of NR iterations should not exceed the given threshold. If it exceeds, set another searching area along the deformation direction and calculate the displacement and strain of the current calculate point by the method in Step 3.

**Step 5**

Repeat Step 4 until the displacement and strain of all calculate points are acquired.

**Figure 4:**
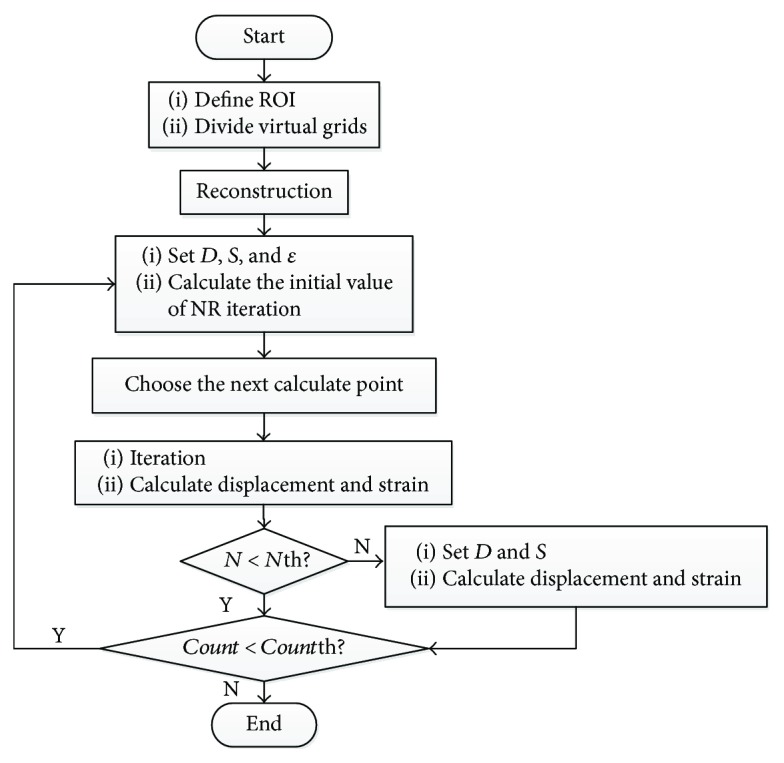
Flowchart of the proposed method process.

The flowchart of the proposed method is shown in Figure 4. *D* is the deformation direction, *S* is the size of the searching area, and *ε*, a very small value, is the precision variable to calculate the reliable displacement and strain for NR iteration. If the difference between the calculated displacement/strain values twice in succession is less than *ε* in an iteration, the convergence conditions of Newton iteration are satisfied. Then, we take the calculated displacement/strain value as the displacement/strain of the current calculate point. *N* represents the number of NR iterations, *N*th represents the threshold of NR iteration number, *Count* represents the number of points whose displacement has been calculated, and *Count*th represents the total of the calculate points.

It is worth noting that the threshold for the NR iteration number *N*th may impact the results. In our method, the NR iteration stops when the number of iterations reaches the threshold or the convergence condition satisfies. If the threshold for the NR iteration number is too small, the NR iteration may stop because of reaching the threshold rather than the iteration convergence, so that the calculated result of the calculate point may be not the best value or not the correct result at all, which would affect the correctness of the next calculate point in turn. So it is necessary to set a suitable value as the threshold for the NR iteration number. Experiments show that when the strain is less than 20%, the NR iteration of each calculate point would satisfy the convergence condition within 20–50 iterations. In this situation, it is proper to set the threshold to 50. When the strain value is between 20% and 50%, the NR iteration of each calculate point would converge within 50–100 iterations. It is more appropriate to set the threshold to 100 under this circumstance. Because the strain values in our experiments range from 0 to 50%, we choose 100 as the threshold for the NR iteration number.

### 2.3. Experiments

#### 2.3.1. Numerical Verification Experiments

Computer-simulated images can provide well-controlled image features and deformation information and are therefore used in our study to verify the accuracy and precision of the proposed method.

Zhou and Goodson [27] proposed an approach to generate simulated speckle images. Speckle patterns before and after deformation are assumed to be the sum of individual Gaussian speckles with random distribution.

(7) $$\begin{array}{c} I_{1}\left(r\right)=\overset{s}{\underset{k=1}{\sum }}I_{0}\;\exp\left(-\frac{{\left|r-r_{k}\right|}^{2}}{a^{2}}\right), \end{array}$$

(8) $$\begin{array}{c} I_{2}\left(r\right)=\overset{s}{\underset{k=1}{\sum }}I_{0}\;\exp\left(-\frac{{\left|r-U\left(r\right)-r_{k}\right|}^{2}}{a^{2}}\right), \end{array}$$

(9) $$\begin{array}{c} U\left(r\right)=U_{0}+\nabla U_{0}r=\left(\begin{array}{l} u_{0}+u_{x}x+u_{y}y\\ v_{0}+v_{x}x+v_{y}y \end{array}\right), \end{array}$$

where *I*_1_ and *I*_2_ denote the reference image and the deformed image, respectively. *s* is the total number of speckles, *I*_0_ is the peak intensity of each speckle, **r**_*k*_ = (*x*_*k*_, *y*_*k*_)^*T*^ is the position of each speckle with a random distribution, and *a* is the speckle size. **U**(**r**) in (9) is the displacement field calculated to describe deformation.

**Figure 5:**
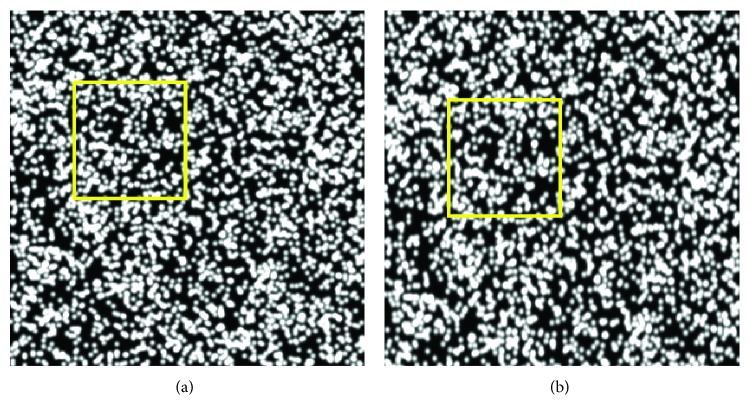
Simulated speckle patterns: (a) reference image; (b) deformed image at the strain of 20%. The imposed yellow rectangle shows the obvious deformation.

In this paper, we used this method to generate ten different strains of speckle patterns, ranging from 5% to 50%, with the increment of 5%.  Figure 5 shows the simulated speckle patterns at the setting strain of 20%. We utilized our proposed method to calculate the strain by reference image and the deformed image and then compared the strain to the setting value to verify the accuracy of our method.

#### 2.3.2. Uniaxial Substrate Stretching Experiments

At present, there are three methods widely used *in vitro* cell-substrate tensile stress experiments: rectangular substrate stretching method, 4-point bending beam loading method, and circular substrate deformation method. The rectangular substrate stretching method is a uniaxial stretching method, which can approximate the mechanical environment of the blood vessel in human body maximally and achieve a more uniform strain [28, 29]. Therefore, uniaxial substrate stretching experiments were performed and our method was applied to measure the strain distribution of the substrate.

**Figure 6:**
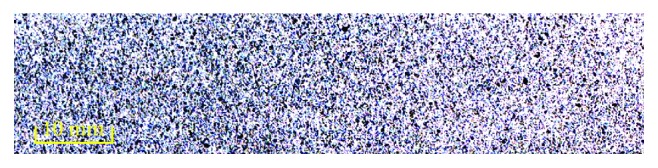
Artificial speckle pattern.

In these experiments, we used medical silica gel film as stretching substrate specimen, which is colorless and transparent. Therefore, the speckle pattern must be artificially made firstly. In this study, black and white quick-drying paints were sprayed on the film (see Figure 6). The size of the substrate specimen was 25 mm × 80 mm (thickness of 0.4 mm).

The stretching device is as Zhou et al. [30] depicted. The substrate was placed between two stainless steel clamps and imaged using an industrial camera (JHSM1400f, Shenzhen Jinghang Technology Co. Ltd.). The images were captured at the selected resolution of 2048 × 1536 pixels. Two stepper motors fixed on an optical platform were used to perform uniaxial stretching. A self-designed multichannel motor control module was used to control these stepper motors. A computer equipped with a 4-port RS-232 USB-to-serial converter (UPort 1450, MOXA) was programmed to provide the accurate synchrony of stepper motors via RS-232 serial ports and the image capture via USB. A series of tension, ranging from 5% to 40% with the increment of 5%, was applied as constant tension. Afterwards, the acquired images were processed with the proposed method to calculate the displacement and strain distribution of the substrate. In these experiments, the central region of substrate, usually as the cell culture area, was selected as the ROI.

## 3. Results and Discussion

### 3.1. Numerical Verification Experiments

**Figure 7:**
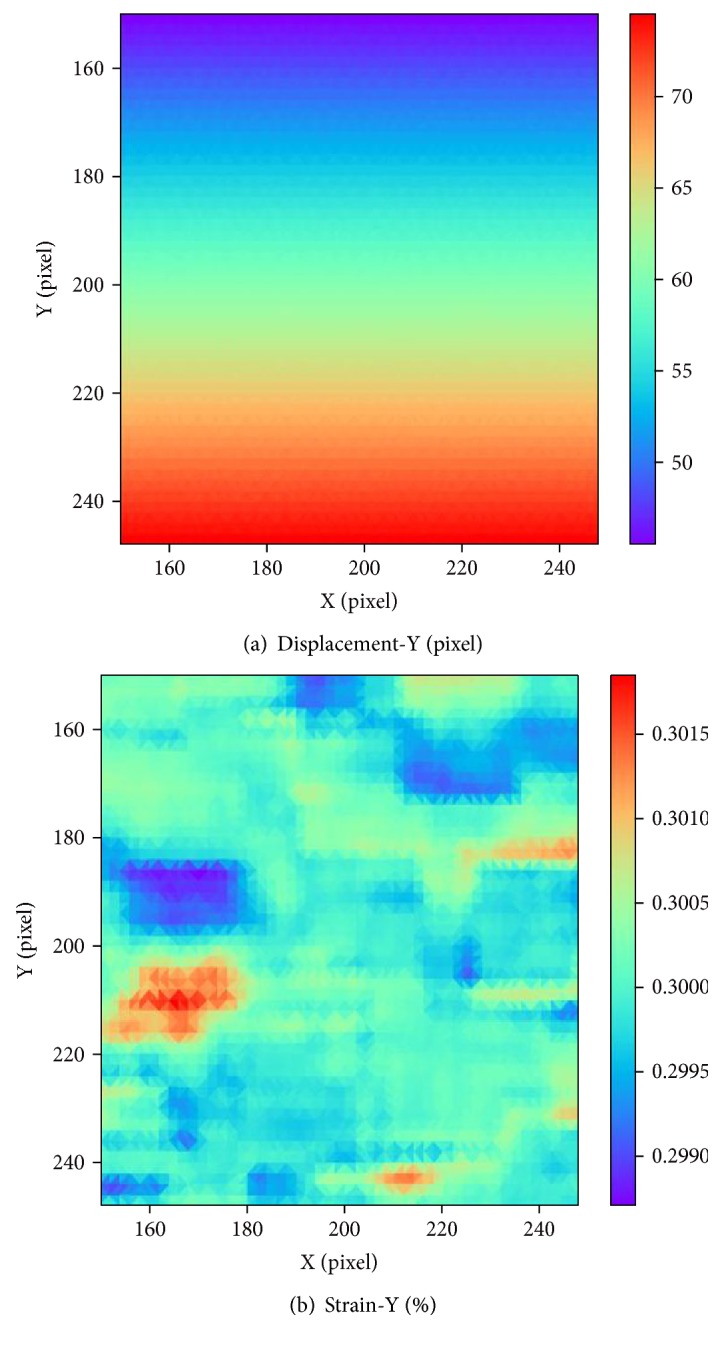
The result of simulated speckle experiment in 30% strain: (a) displacement distribution along *y*-axis; (b) strain distribution along *y*-axis.

In the simulation experiment, normal strain was exerted in *y* direction in the value of 5%, 10%, 15%, 20%, 25%, 30%, 35%, 40%, 45%, and 50%. In our method, the displacement and strain distribution can be acquired.  Figure 7 shows the calculation result at the strain of 30%. The horizontal coordinate is the position in *x* direction, and the vertical coordinate is the position in *y* direction. Different colors in legend represent different displacement values or strain values. As shown in Figure 7, the displacement field is continuous and the strain distribution is generally uniform with small standard deviations. The average of measuring strain is 30.00083%, which is very close to the setting value 30%, demonstrating that the proposed method can measure the large strain accurately.

All results of these simulated speckle experiments are displayed in Table 1. Setting strain is the parameter used to generate deformed images. Computing strain, presented as mean ± standard deviation, denotes the calculation value by our proposed method. According to Table 1, the computing strains in all images are very close to the setting stains with small bias error. Additionally, each computing strain has small standard deviation. These results suggest that the proposed method is highly accurate and can be used in calculating the strain distribution under large deformation.

### 3.2. Uniaxial Substrate Stretching Experiments

**Figure 8:**
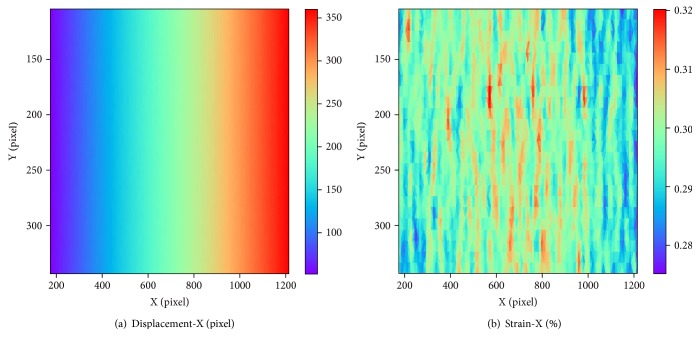
The result of uniaxial substrate stretching experiments at the tension of 30%: (a) displacement distribution along *x*-axis; (b) strain distribution along *x* direction.

Figure 8 shows the calculation results at the tension of 30% by the proposed method. The horizontal and vertical coordinate is the position in *x* direction and *y* direction, respectively. Different colors in legend represent different displacement value or strain value. The displacement is continuous and changes along *x* direction as expected. The strain values are ranging from 28% to 32% and concentrate at 30%.

**Figure 9:**
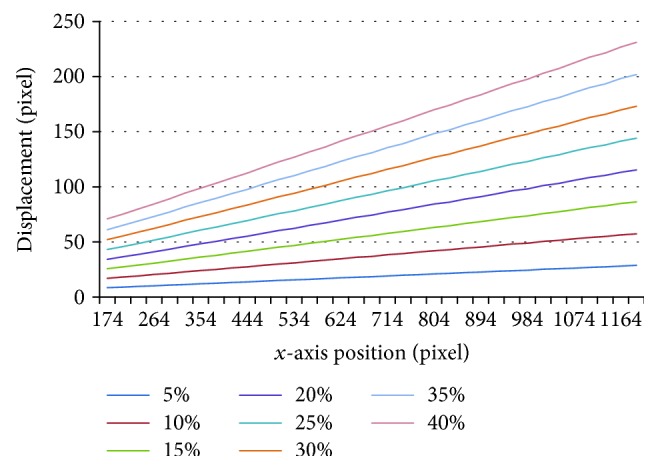
The distribution of displacement under different tension along *y* direction.

**Figure 10:**
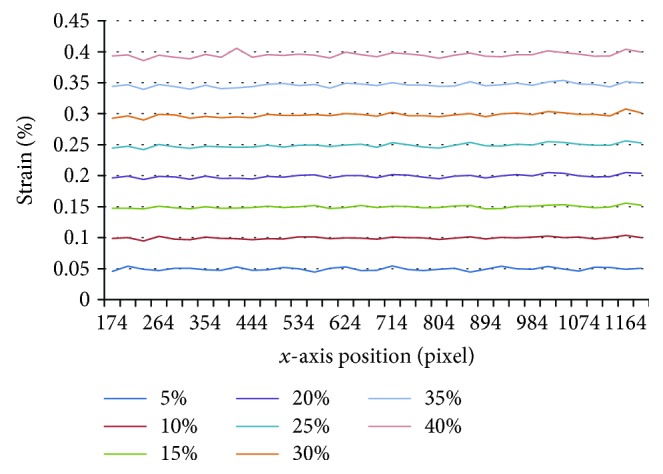
The distribution of strain under different tension along *y* direction.

As the tension was loaded in the *x* direction, we evaluated the component of the displacement and strain vector in the *x* direction, and the results can be seen in Figures 9 and 10.  Figure 9 shows the distribution of displacement under different tension along *x*-axis. The horizontal axis represents the position in *x* direction, and the vertical axis represents the displacement of substrate calculated by the proposed method along *x* direction under different tension. Different colors represent different strain group. The displacement along *x*-axis appears the same rising tendency at every step and also increased with tension.  Figure 10 shows the distribution of strain under different tension along *x*-axis. The horizontal axis represents the position in *x* direction, and the vertical axis represents the strain of substrate calculated by the proposed method along *x* direction under different tension. Different colors represent different strain group. The strains along *x*-axis remain stable and appear small fluctuation in each tension, indicating that the substrate is homogeneous.

We compared the strains calculated by the proposed method with which obtained from the stretching equipment, the result is shown in Table 2. Reference strain is the strain calculated by the displacement from the stretching equipment and the initial length of the substrate. Measured strain, presented as mean ± standard deviation, is calculated by the proposed method in this paper, which is the average value of strain field in the central region of substrate sized 1050 × 250 pixels. The relative error equals the ratio of the difference between the reference strain and the measured strain to the reference strain.

According to Table 2, the measured strain is always very close to the reference strain. The differences between the reference strains and the calculated strains are tiny, and the maximum of relative errors is 1.75%, which shows that the proposed method can be used for large strain measurement in the actual tensile experiments. Moreover, every measured strain appears small standard deviation, demonstrating that the strain distribution of the substrate is uniform and the substrate is homogeneous.

## 4. Conclusions

In order to obtain accurate strain distribution *in vitro* mechanical stretching of cell culture, an improved method is proposed in this paper and combined with DICe. The proposed method successfully solved the problem that DICe could not be used directly to compute the large strain. To evaluate the effect and accuracy of the proposed method, numerical experiments were performed. The results demonstrated that accurate displacement and strain field could be acquired by the proposed method. Moreover, uniaxial substrate stretching experiments were performed and the proposed method was used for strain distribution measurement of the substrate, large strain in the actual tensile experiments to obtain accurate strain distribution. In addition, our method can be applied to measure strain in a wide range, especially in strain distribution measurement of soft tissues. Combining our method with 3D DIC technique, the 3D displacement field and surface strain field of 3D object can be measured. It can be applied to soft tissue deformation measurement (e.g., muscles and skin) in mechanobiology and sports medicine experiments. Thus, it could allow experimenters to adequately research the response of cells and soft tissues to different strains.

## Figures and Tables

**Table 1:** Comparison between setting strain and computing strain.

Setting strain (10^−2^)	Computing strain (10^−2^)	Bias error
5	5.001 ± 0.037	1.0 *e*−05
10	10.002 ± 0.038	2.0 *e*−05
15	15.002 ± 0.047	2.0 *e*−05
20	20.004 ± 0.047	4.0 *e*−05
25	24.999 ± 0.045	1.0 *e*−05
30	30.001 ± 0.044	1.0 *e*−05
35	35.003 ± 0.044	3.0 *e*−05
40	40.000 ± 0.039	0
45	45.004 ± 0.041	4.0 *e*−05
50	49.998 ± 0.044	2.0 *e*−05

**Table 2:** Comparison between the proposed method and stretching equipment.

Reference strain (%)	Measured strain (%)	Relative error (%)
5	4.95 ± 0.59	1.00
10	9.97 ± 0.52	0.30
15	15.0 ± 0.58	0.00
20	19.9 ± 0.67	0.50
25	24.9 ± 0.73	0.40
30	29.8 ± 0.78	0.67
35	34.6 ± 0.85	1.14
40	39.3 ± 1.13	1.75
